# Breast cancer and physical activity: A bibliometric analysis

**DOI:** 10.3389/fonc.2022.1051482

**Published:** 2023-01-12

**Authors:** S. Fresno-Alba, Á. Denche-Zamorano, R. Pastor-Cisneros, D. Pereira-Payo, J. M. Franco-García, R. Jiménez-Castuera

**Affiliations:** ^1^ Didactic and Behavioral Analysis in Sport Research Group, Faculty of Sport Sciences, University of Extremadura, Cáceres, Spain; ^2^ Promoting a Healthy Society Research Group (PHeSO), Faculty of Sport Sciences, University of Extremadura, Cáceres, Spain; ^3^ Health Economy Motricity and Education (HEME), Faculty of Sport Sciences, University of Extremadura, Cáceres, Spain

**Keywords:** breast cancer, physical activity, quality of life, health, women

## Abstract

Breast cancer is a pathology suffered by millions of women worldwide, affecting the mental health, quality of life (QoL), physical functioning, cognitive functioning, and social and work life of surviving patients. Moreover, breast cancer is associated with weight gain, muscle atrophy, and weakness. Physical exercise appears to be an effective non-pharmacological treatment to improve short-term self-esteem, QoL, fatigue, and psychological factors such as depression, anxiety, happiness, and body image. The practice of physical activity is also associated with a reduction in the side effects of treatment. This bibliometric analysis analyzed the trend followed by publications on breast cancer and physical activity. The Web of Science database was used, and bibliometric laws were applied to identify the most prolific authors, the journals most involved in the field, and the countries, institutions, and keywords most used by the authors. Breast cancer and physical activity have an exponential trend in the number of publications, with *Psycho-Oncology* being the journal with the highest number of publications.

## 1 Introduction

Breast cancer (BC) is a cancer type commonly diagnosed in women. It is the fifth leading cause of cancer death in the world (1) and the leading cause of cancer death in women (2). Globally, BC has increased its prevalence by approximately 2.3 million new cases and has a mortality rate of 6.9% (1). In Spain, it is suffered by 0.12% of the female population aged 15 years and above, 0.10% between 15 and 65 years, and 0.24% of women over 65 years, with an increase between 2012 and 2019 of 7.5% (3). In Spain, the economic cost for BC was approximately 44 million Euros (4). Due to the increased prevalence of risk factors, early detection by mammography, and aging, it has been possible to observe a higher incidence of BC (5). In contrast, the mortality rate is decreasing, which indicates significant advances in cancer therapy (6).

BC risk factors can be classified in these categories: modifiable, non-modifiable, and protective. The main non-modifiable risk factors are as follows: age (increased incidence after 35 years and stabilization at 55 years), family medical history (7), and hormonal status (8). The modifiable risk factors are as follows: having given birth (reduces the risk by 10%) (8), breastfeeding (reduces the risk by 2% for every 5 months of breastfeeding) (9), and alcohol consumption (increases the risk by up to 30%) (10). Finally, regarding protective factors, physical activity (PA) (11), nutrition (12), and screening (13) have been related as protective factors for BC in postmenopausal women, although the evidence is limited.

On the other hand, once the disease is present, the main treatments for BC are systemic treatments (hormone therapy, chemotherapy, and biological agents) combined or not with local treatments such as surgery and radiotherapy (14).

In addition, cancer is associated with serious complications such as muscle atrophy, weakness, and weight gain due to a poor QoL (15, 16). These adverse sequels are also a consequence of physical inactivity, which decreases physical function, aerobic capacity, and QoL (15). In this regard, there is increasing scientific evidence of the physical and psychological benefits of exercise during and after BC treatment (17). Various studies (18–21) show that exercise can have positive physical and psychological effects for the patient during treatment (improvements in aerobic fitness, upper and lower body strength, body weight, fat percentage, QoL, mood, anxiety, self-esteem, increased treatment efficacy, immunity, and bone health) and after treatment (those mentioned above, in addition to improvements in body mass index, level of physical exercise, fatigue, general symptoms and side effects, relapses, survival rate, and life expectancy) (18–21). Consequently, the practice of PA is associated not only with an increase in post-diagnostic QoL but also with a reduction in the side effects of treatment (22, 23). Thus, there is evidence that the practice of regular PA, following the recommended guidelines, decreases the risk of recurrence and mortality of cancer patients before diagnosis and during and after treatment (24).

Phillips and McAuley (23) exhibited the importance of exercise on QoL, fatigue, and other psychosocial factors such as anxiety, depression, self-esteem, happiness, and body image in women with BC (23). Consistent with this fact, there is significant evidence demonstrating an increased risk of anxiety, depression and suicide, and neurocognitive and sexual dysfunction in BC survivors compared to women without a previous cancer diagnosis (25).

A bibliometric study aims to analyze the scientific interest and evolution of a given field of knowledge, evaluating the annual publications, most prolific and prominent authors, institutions and countries concerned, or most cited articles. The present study, more specifically, will be the first to address scientific papers published on the following topics: BC, PA, and mental health.

Thus, the temporal evolution of studies on BC in relation to PA and mental health presented by diagnosed patients and/or survivors makes it necessary to carry out a broad meta-analytical and current study. This study will provide a panoramic view of the scientific and practical community, feasible through a bibliometric approach that analyzes the data and metadata of specific preexisting articles on this topic. So, the main aims are to analyze the trend of annual publications, to identify the most prolific and cited journals and authors, and to highlight the most used keywords and the most relevant articles.

## 2 Materials and methods

### 2.1 Design

This research followed the design of a descriptive bibliometric analysis to perform a scientific mapping of scientific publications on PA and BC similar to those used in other research (26–29).

### 2.2 Source of data

The Web of Science (WoS) Core Collection (WoCSS) database of Clarivate Analytics was used as a data source. This database is one of the most widely used by researchers to perform bibliometric analyses, given its wide catalog of prestigious indexed journals, its influence in the scientific field, and the complete information provided for this type of analysis (30, 31).

Data were extracted from WoSCC on 27 June 2022. The search was performed as follows: (ti=(“breast cancer”) or ti=(“breast lymphoma”) or ti=(“breast neopl*”) or ti=(“breast tumou*”) or ti=(“ductal carcinoma in situ”) or ti=(“invasive ductual carcinoma”) or ti=(“lobular carcinoma in situ”) or ti=(“invasive lobular cancer”) or ti=(tnbc) or ti=(“breast carci*”) or (ti=(carcinoma) and ti=(breast))) and (ts=(“physical activity”) or ts=(exercise)) and (ts=(“mental health”) or ts=(anxiety) or ts=(depression)) and (dt=(“article” or “review”)). The search was restricted to the editions: Science Citation Index Expanded (SCI-EXPANDED), Social Sciences Citation Index (SSCI), and Emerging Sources Citation Index (ESCI), limiting the search to articles and reviews, with no other temporal, language, or design limitations. In WoS, the “ti” tag is used to search for articles that contain a given concept in the title, while the “ts” tag is used to search for articles containing a given concept in the topic (title, abstract, keywords, and keywords plus). The dataset was downloaded in “Plain text file” format and then analyzed with Microsoft Excel (version 2206; Microsoft Corporation, Redmond, WA, USA) and VOSviewer (Center for Science and Technology Studies, Netherlands) software.

### 2.3 Statistical analysis

For the scientific mapping of the object of study, a descriptive bibliometric analysis was performed. Price’s exponential growth law (32, 33) was applied, analyzing the distribution of annual publications on the subject up to the year 2021 (the year 2022 was excluded, as it did not present complete data) and whether they were in a phase of exponential growth, which was evaluated with the coefficient of determination (R^2^). An exponential growth of the annual number of publications ensures interest in the subject and a broad base of researchers working on its development (34). The journals more interested in the topic and those that accumulated the highest number of citations were analyzed by applying Bradford’s law of concentration of science, identifying the most prolific journals and the most cited (34–39), providing researchers with information to locate and select the most productive and relevant journals that are publishing in a particular area of knowledge (40). The most productive and cited journals (Bradford’s core) were presented, providing quartile in which journals were ranked in WoS, number of documents in the object of study of this research, and Open Access percentage of its articles, among others. A descriptive analysis of the countries/regions that co-authored the publications was performed, indicating the most prolific countries, as well as the countries with the highest number of citations, allowing for the examination of cooperation between countries and reflects stronger social links than other measures of relevance (41, 42). Lotka’s law was applied to identify the most prolific co-authors (43); this allowed the most productive authors or occasional researchers to be identified (36) and how they published among themselves, learning about production networks. In order to highlight the co-authors who accumulated the most citations among articles analyzed, the Hirsch index (h-index) (44) was applied, considering the most influential co-authors to be the h co-authors with h number of citations among the articles analyzed. The h-index was also used to highlight the most cited documents, considering these as the most influential (28) in the subject (37). The words of greatest interest to the researchers were identified by applying Zipf’s law to the author keywords used in the set of articles (45). For visualization graphs, the association strength and fractionalization analyses performed by the VOSviewer software were used. Type of analysis used: Journals (Citation: The relatedness of items is determined based on the number of times they cite each other); Authors and Countries/Regions (Co-authorship: The relatedness of items is determined based on their number of co-authored documents); Author’s Keywords [Co-occurrences: The relatedness of items is determined based on the number of documents in which they occur together (46–49)]. More information about this analysis, node size, strength links, scores, and density maps, among others, could be found in the VOSviewer Manual.

## 3 Results

### 3.1 Exponential growth in annual publications

**Figure 1:**
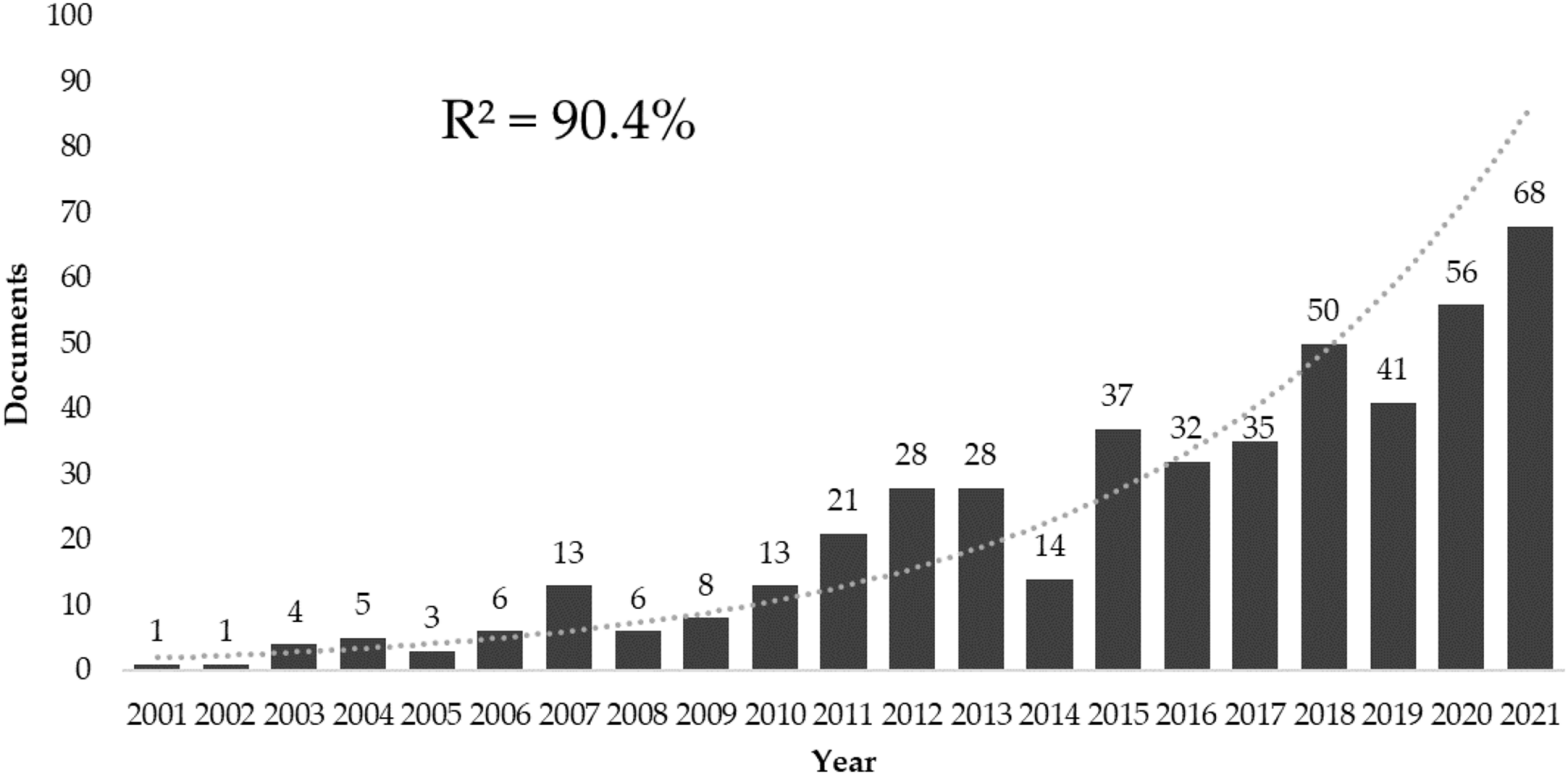
Annual production of research on breast cancer and physical activity.

A total of 506 documents were collected: 442 articles and 64 review articles published between 1991 and 2022. No continuity was found in the annual number of publications up to 2001, so it was decided to analyze the exponential growth of publications between 2001 and 2021 (**Figure 1**), excluding the previous years, and the current year, as it did not present complete data.

### 3.2 Most prolific and most co-cited journals

**Figure 2:**
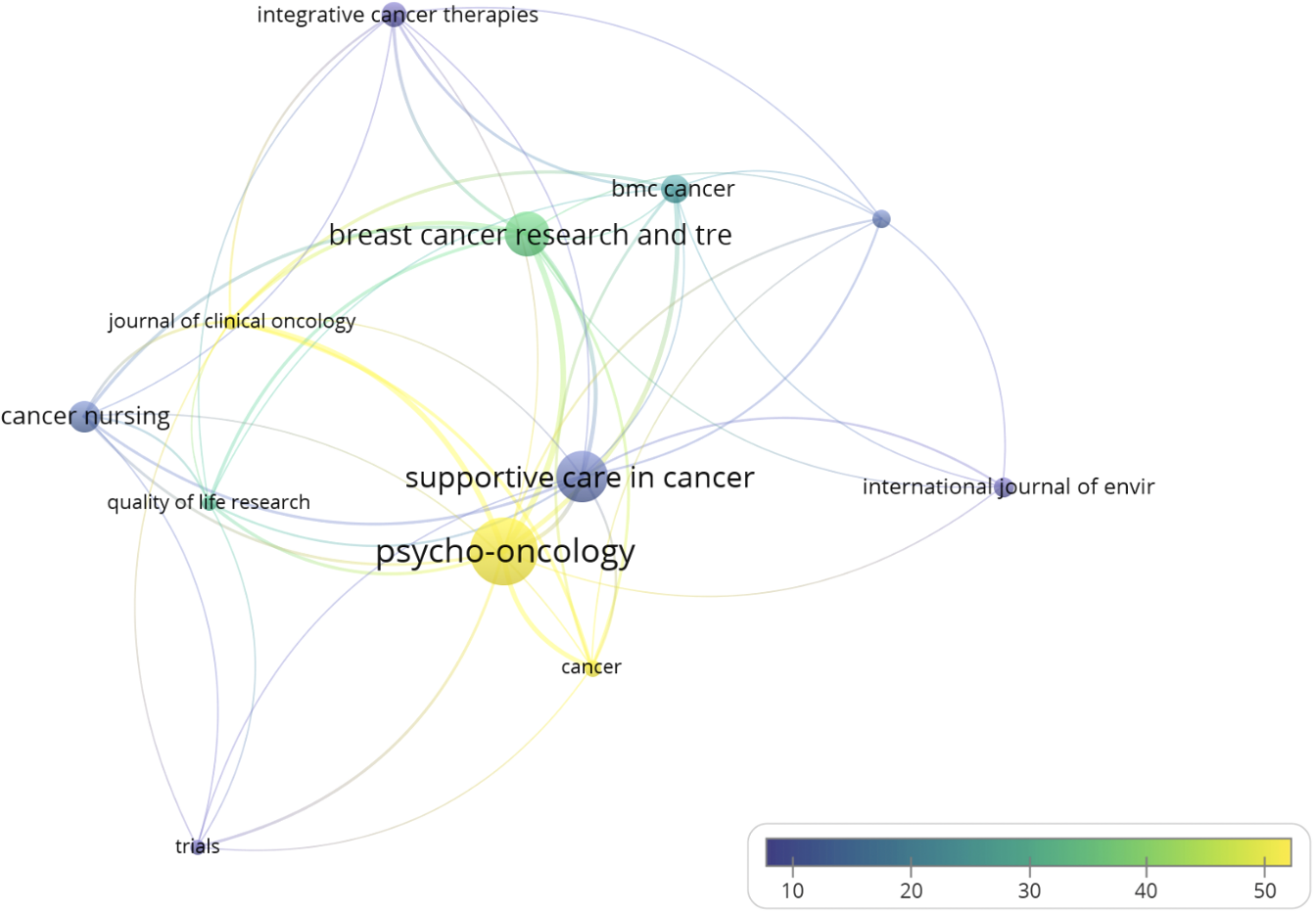
Core of journals.

The results of the analysis of publication sources showed a total of 221 journals. The journal with the most publications was *Psycho-Oncology* (36 papers, 7.1% of total publications), published by Wiley, followed by *Supportive Care in Cancer* (27 papers, 5.3%). **Table S1** shows the 12 journals that formed the core of the most productive journals in the study topic of this research according to Bradford’s zones (**Table S2**); these journals accumulated 36.8% of all publications. Fifty percent of the journals were in the first quartile of one of the WoS categories. **Figure 2** shows the 12 most prolific journals and their interrelationships. The size of the node corresponds to the number of documents per journal, the size of the connections to the link strength, and the color to the mean number of citations per article of each journal (Analysis: Fractionalization; Attraction: 10; Repulsion: 0).

According to the number of citations, Bradford’s core for the journals was composed of four journals (**Table S3**), *Psycho-Oncology* (36 documents, 1,792 citations), *Journal of Clinical Oncology* (8 documents, 1,416 citations), *Breast Cancer Research and Treatment* (24 documents, 841 citations), and *Journal of Experimental & Clinical Cancer Research* (1 document, 466 citations), accumulating 33.2% of the citations (**Table 1**).

**Table 1:** Bradford core for journals on physical activity and breast cancer according to the number of citations of articles published in the journals.

Bradford’s zone	Journals (Publisher)	JCR	Articles	Citations	% Citations	% Acc.	% OA
**Core**	Psycho-Oncology (Wiley)	Q1	36	1792	13.2%	13.2%	10.5%
	Journal of clinical oncology (Lippincott Williams & Wilkins)	Q1	8	1416	10.4%	23.6%	15.9%
	Breast cancer research and treatment (Springer)	Q2	24	841	6.2%	29.8%	21.4%
	Journal of experimental & clinical cancer research (BMC)	Q1	1	466	3.4%	33.2%	99.8%

% Acc., percentage of accumulated cites; % OA, percentage of open access.

### 3.3 Countries/regions

**Figure 3:**
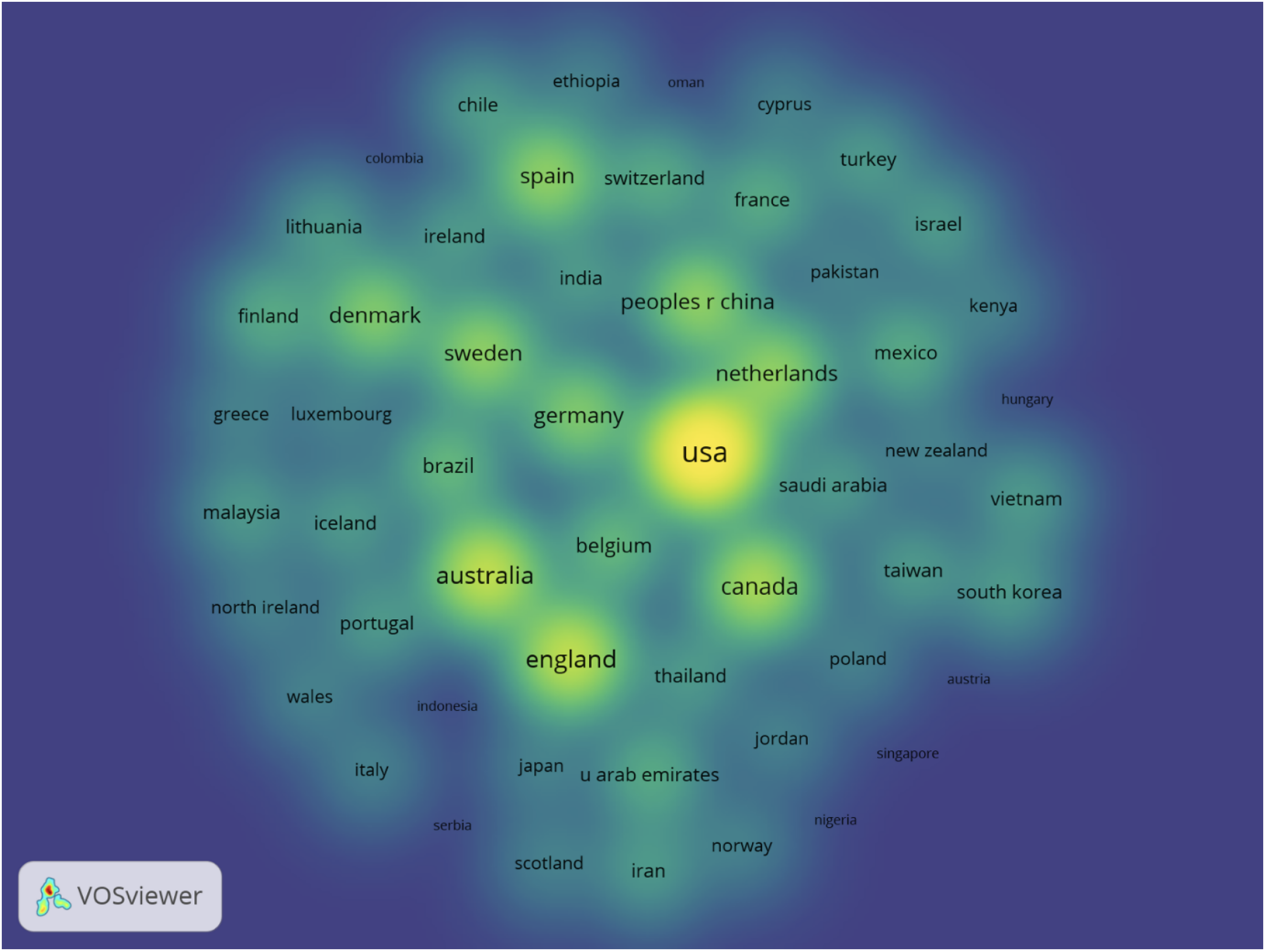
Density map of countries in co-authorship.

The 506 papers were co-authored by researchers affiliated with institutions in 55 countries/regions (**Table S4**). The largest number of publications were co-authored in the United States (190 papers, 37.6%), Canada (55 publications, 10.9%), and China (41 papers, 8.1%). **Figure 3** shows the density map of countries in co-authorship, the size of word, and circumference; the opacity of yellow is positively related to the number of documents.

**Figure 4:**
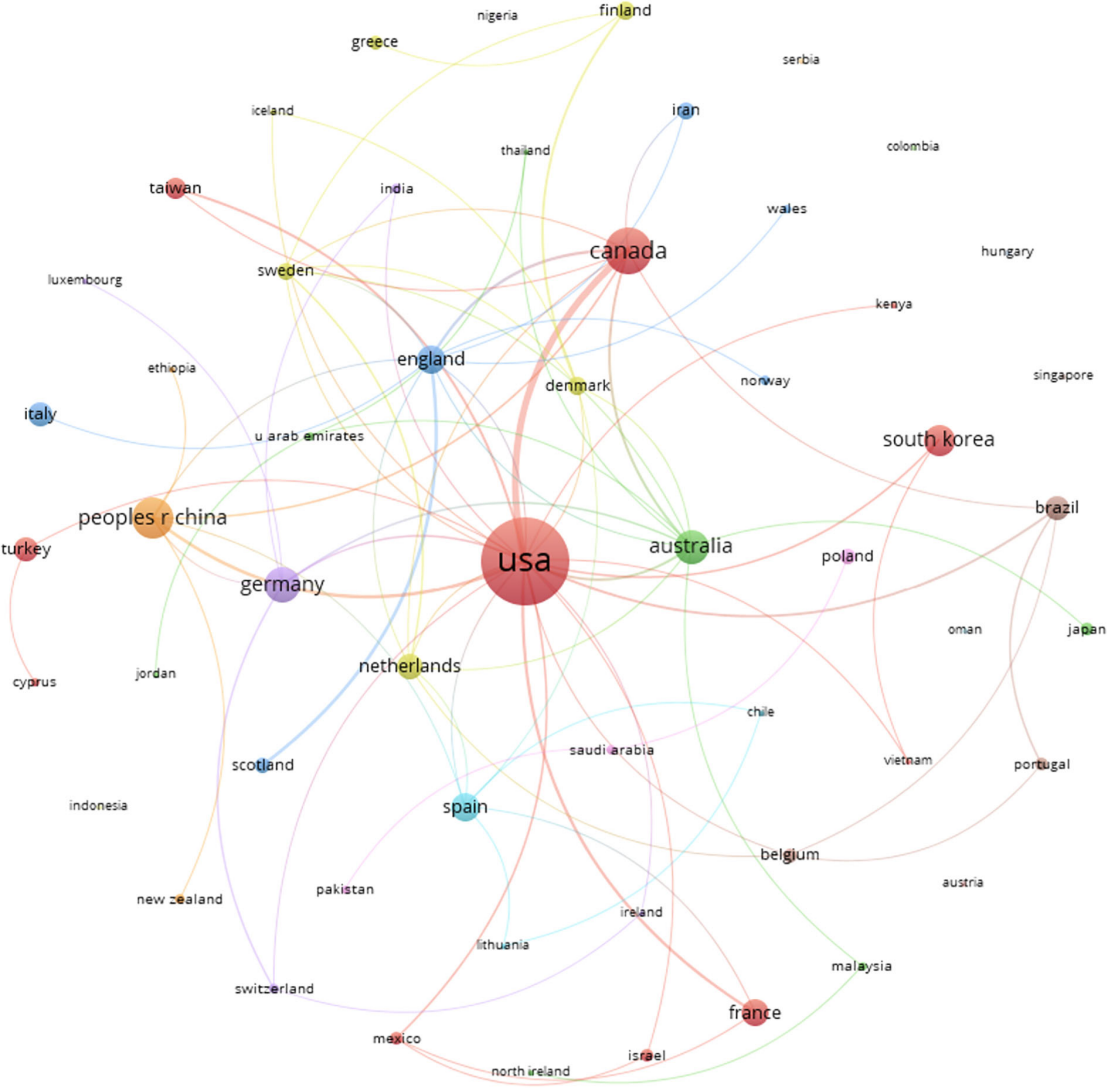
Countries in co-authorship and their interrelationships.

The country with the most co-citations was also the United States (5,408 citations, 39.8%), followed by Canada (2,465 citations, 18.1%) and Germany (1,036 citations, 7.7%). **Table S4** shows the co-authored countries ranked according to the number of papers published with co-authors from that country and the number of citations accumulated by these co-authors. **Figure 4** shows the 55 countries/regions and their interrelationships. Node size corresponded to the number of documents, the clusters formed by the countries were represented by colors, and the thickness of the lines represented the link strength (number of links) (Analysis: Associations strength; Attraction: 9; Repulsion: -1; Minimum cluster size: 3). The United States led the largest and most productive cluster (Cluster Red: 11 countries/regions), publishing with authors from Canada, Cyprus, France, Israel, Kenya, Mexico, South Korea, Taiwan, Turkey, and Vietnam, with another large and productive cluster led by Australia, publishing with Japan, Jordan, Malaysia, North Ireland, Thailand, and United Arab Emirates (Cluster Green: seven countries/regions). The United States (23 unique collaborations) was the country with the highest number of collaborations with other countries/regions, followed by Australia (11 unique collaborations) and England (11 unique collaborations).

### 3.4 Co-authors and co-author co-citations

**Figure 5:**
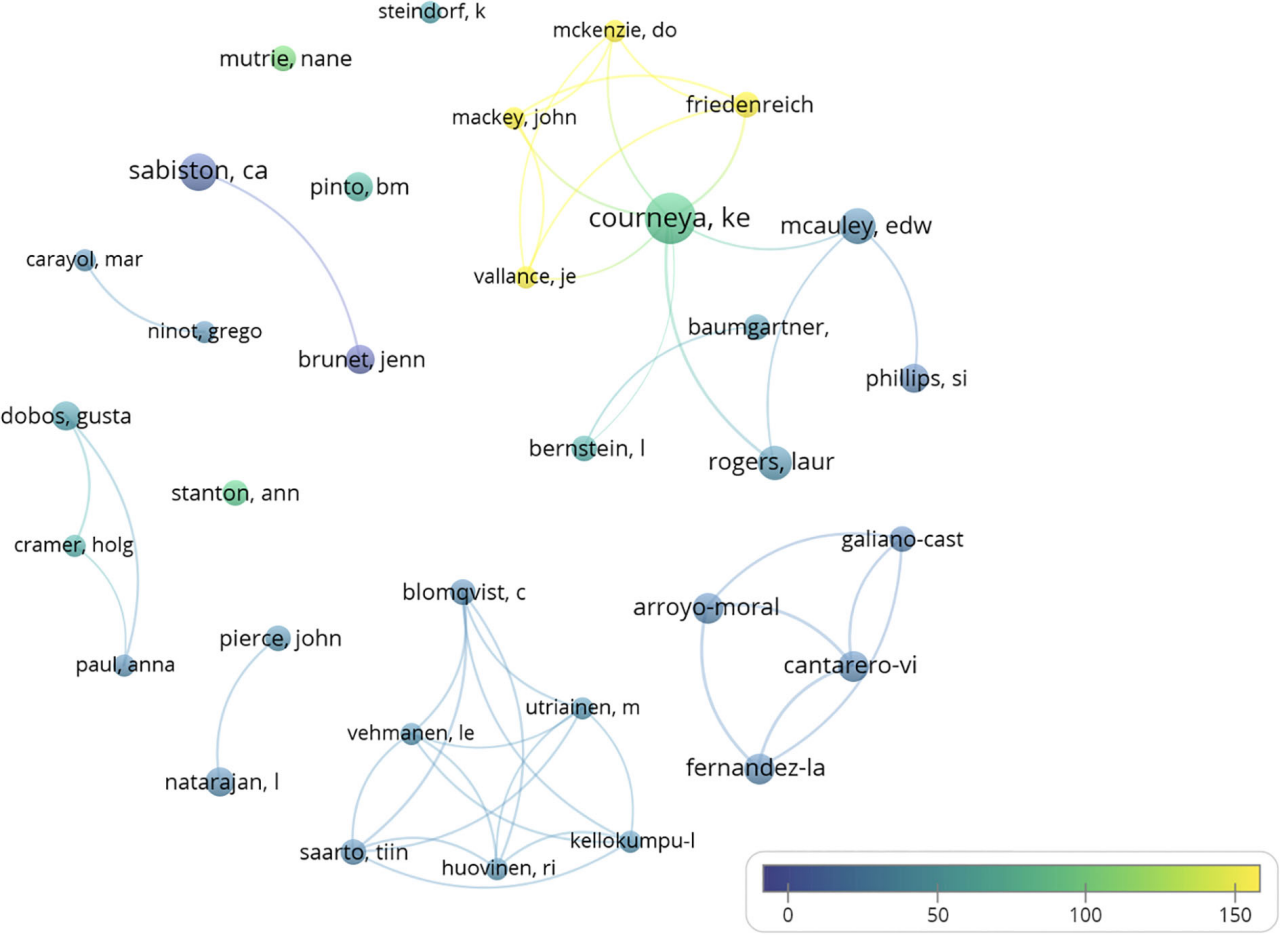
Graph with the most relevant co-authors.

A total of 2,690 co-authors were found, with a range of co-publications between 1 and 17 documents (**Figure S1**. Histogram of co-authors by number of publications). Only two co-authors were found with more than 10 papers: Courneya (17) and Sabiston (11). A total of 2,265 co-authors had published only one paper at the time of analysis. Applying Lotka’s law, it was estimated that the number of most prolific co-authors should be equal to or less than 52 (square root of 2,690). We found 78 co-authors with four or more papers and 34 co-authors with five or more papers, making these the most prolific co-authors (**Table S5**). Among these authors, the h-index was applied, looking for the n authors with n citations, considering these as the most prominent. Thirty-three authors were found, with at least 33 citations. **Figure 5** shows the most relevant co-authors and their interrelationships (Analysis: Fractionalization; Attraction: 8; Repulsion: -1; Node size: Number of publications; Thickness of connections: Link Strength; Color: Average number of citations per article). Different groups of collaboration between authors were found. The most prolific group, formed by nine co-authors, was led, in terms of number of publications, by Courneya.

**Figure 6:**
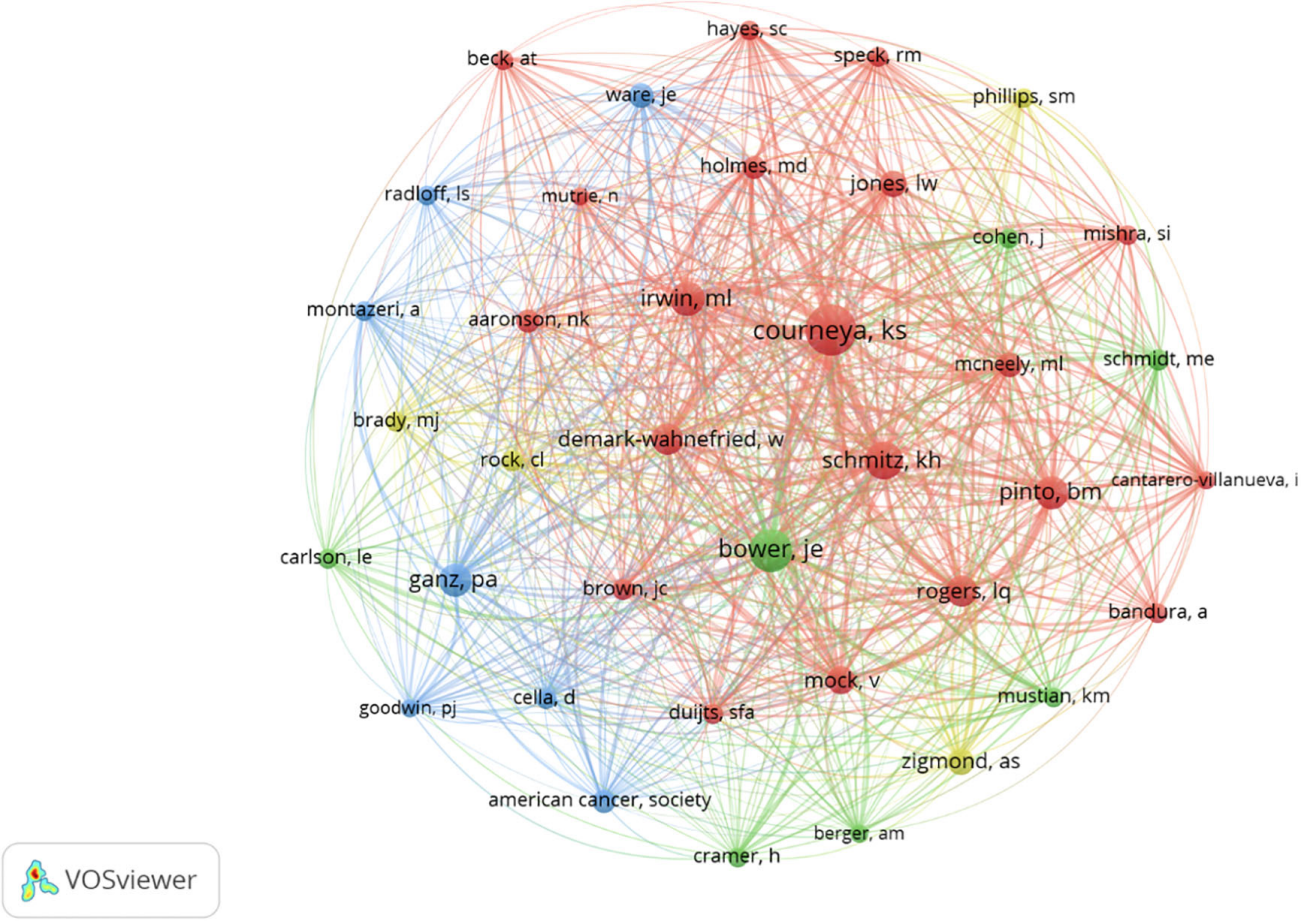
Graph with the most co-citated co-authors.

We found 11,241 co-cited co-authors, authors who have been co-cited together in at least one publication. Following the number of most prolific co-authors, the 34 co-authors with the highest number of co-citations were highlighted; these authors were co-cited at least 41 times. Courneya was the co-author with the highest number of co-citations (280). **Figure 6** shows the graph of co-citations.

### 3.5 Most cited articles

Applying the h-index to the articles, a total of 60 articles with at least 60 citations were obtained (Annex 1). **Table 2** shows the 10 articles with the highest number of citations, being “Effects of aerobic and resistance exercise in breast cancer patients receiving adjuvant chemotherapy: A multicenter randomized controlled trial,” by Couerneya, the most cited article, with 663 citations. Within the 10 most cited documents, six were articles and four were reviews, all of them written in English.

**Table 2:** The 10 most cited articles.

Main Author	Article Title	Citations	Journal ISO Abbreviation	Language	Document Type
Courneya, K. (50)	Effects of aerobic and resistance exercise in breast cancer patients receiving adjuvant chemotherapy: A multicenter randomized controlled trial	663	J. Clin. Oncol.	English	Article
Montazeri, A. (2008) (51)	Health-related quality of life in breast cancer patients: A bibliographic review of the literature from 1974 to 2007	466	J. Exp. Clin. Cancer Res.	English	Review
Howard-Anderson, J. (2012) (52)	Quality of Life, Fertility Concerns, and Behavioral Health Outcomes in Younger Breast Cancer Survivors: A Systematic Review	446	JNCI-J. Natl. Cancer Inst.	English	Review
Duijts, S. (2011) (53)	Effectiveness of behavioral techniques and physical exercise on psychosocial functioning and health-related quality of life in breast cancer patients and survivors—A meta-analysis	266	Psycho-Oncol.	English	Review
Mutrie, N. (2007) (54)	Benefits of supervised group exercise programme for women being treated for early stage breast cancer: Pragmatic randomised controlled trial	258	BMJ-British Medical Journal	English	Article
McKenzie, D. (2003) (55)	Effect of upper extremity exercise on secondary lymphedema in breast cancer patients: A pilot study	205	J. Clin. Oncol.	English	Article
Daley, A. (2007) (56)	Randomized trial of exercise therapy in women treated for breast cancer	194	J. Clin. Oncol.	English	Article
Culos-Reed, S. (2006) (57)	A pilot study of yoga for breast cancer survivors: Physical and psychological benefits	183	Psycho-Oncol.	English	Article
Furmaniak, A. (2014) (58)	Exercise for women receiving adjuvant therapy for breast cancer	159	Cochrane Database Syst Rev.	English	Review
Kiecolt-Glaser, J. (2014) (59)	Yoga’s Impact on Inflammation, Mood, and Fatigue in Breast Cancer Survivors: A Randomized Controlled Trial.	155	J. Clin. Oncol.	English	Article

### 3.6 Most used keywords

**Figure 7:**
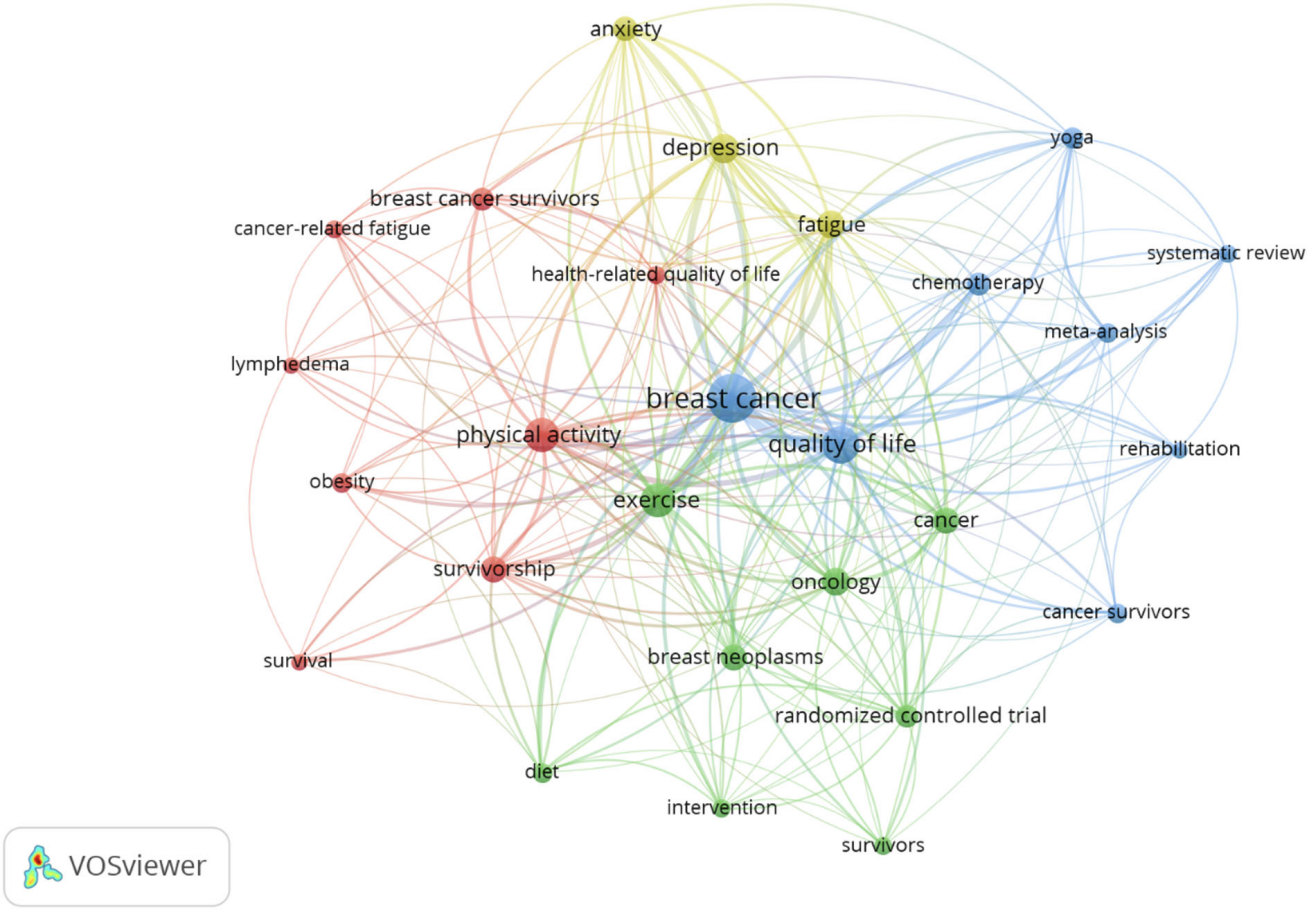
Graph with the most used keywords by authors.

By applying Zipf’s law to the author keywords, 27 terms were highlighted. **Figure 7** shows the most used keywords by authors using a co-occurrence analysis with VOSviewer (Analysis: Association strength; Attraction: 2; Repulsion: 0; Minimum cluster size: 3; Node size as a function of the number of occurrences; Thickness of connections: Link strength; Color: Cluster formed). In the association strength analysis, four clusters were found; the terms breast cancer (266 uses) and QoL (115 uses) were the most used keywords, forming a cluster (blue) with other terms such as chemotherapy (22 uses) and cancer survivors (15 uses). Another cluster (red) was formed with terms such as physical activity (85 uses) and survivors (40 uses), together with others such as breast cancer survivors (26 uses) or obesity (15 uses). The term exercise (87 uses) formed a cluster with terms such as oncology (42 uses), cancer (34 uses), or breast neoplasia (34 uses). Finally, a cluster was found formed by the terms fatigue (49 uses), depression (58 uses), and anxiety (31 uses).

**Figure 8:**
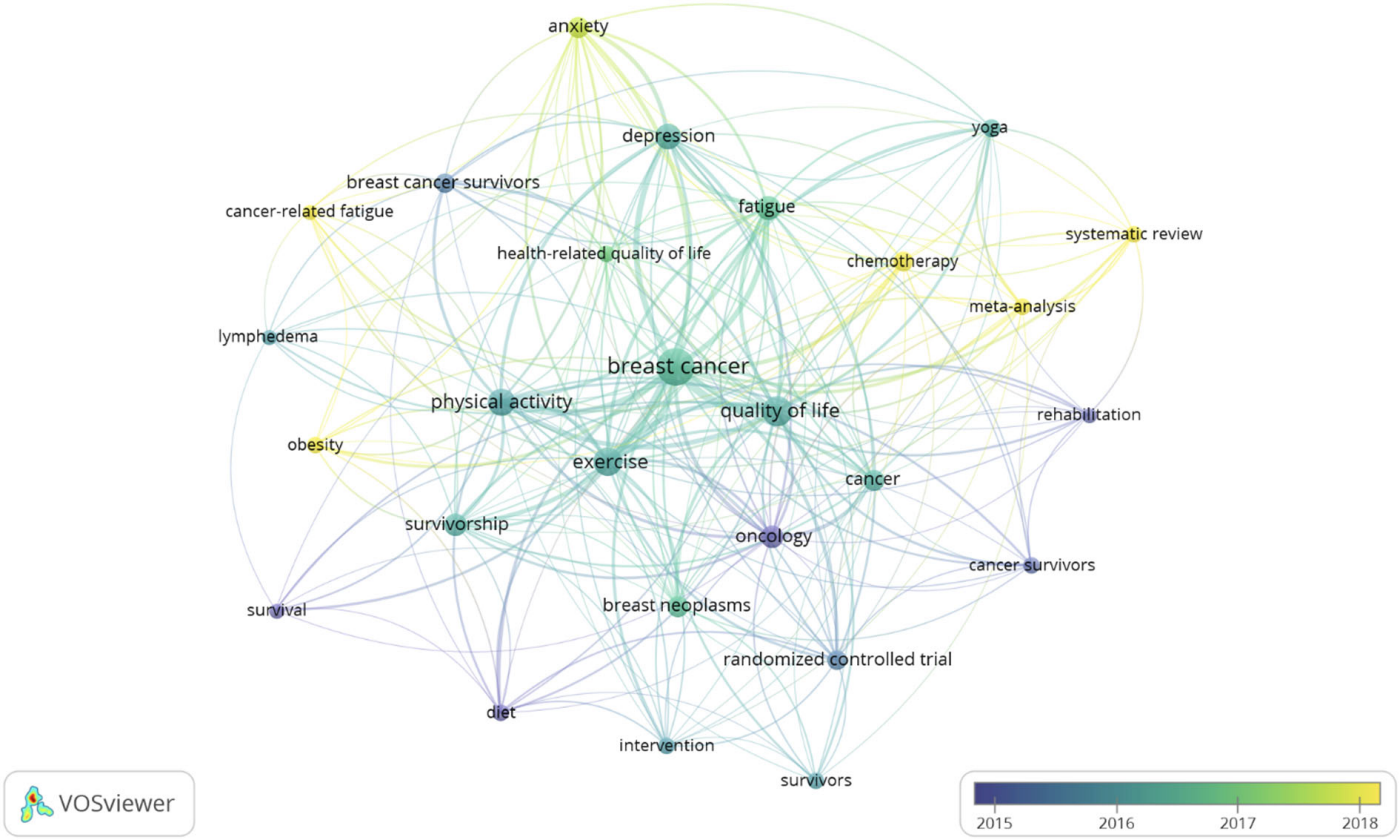
Graph with the most used author keywords: average publication years.

**Figure 8** shows the graph with the keywords as a function of the average years of publications in which they appeared (Analysis: Association strength; Attraction: 2; Repulsion: 0; Node size as a function of the number of occurrences; Linkage thickness: Link strength; Color: mean year of publication). Terms such as obesity, chemotherapy, anxiety, or self-reported fatigue were the terms with the most recent mean years of publication.

## 4 Discussion

This bibliometry analyzed the properties of 506 documents, more specifically, 442 individual studies and 64 review articles on BC and PA published from 1991 to 2021 in WoSCC. There have been 2,690 co-authors from 55 countries/regions who published on this topic, and in this time range, the most prolific regions are the United States, Canada, and China. The first co-author to initiate research on this topic was Ellen Levine in 1991. However, an exponential growth in the number of publications was not observed until 2001, as no continuity was found in previous years. Additionally, in the years 2008 and 2014, the increasing pace of the number of publications slowed down, finding the period of maximum peak and growth between 2018 and 2021, which is worth noting, since the number of publications could be expected to decrease due to the coronavirus disease (COVID) pandemic.

The present study analyzed a total of 221 journals, among which *Psycho-Oncology* stands out as the journal that publishes most articles on this subject, followed by *Supportive Care in Cancer*. However, if we look at the number of citations of the articles published in each journal, as shown in **Table 1**, there is *Psycho-Oncology* (36 articles) in the first position, and behind it, the journals that stand out more are as follows: *Journal of Clinical Oncology*, *Breast Cancer Research and Treatment*, and *Journal of Experimental & Clinical Cancer Research*. *Psycho-Oncology* is a journal with multidisciplinary interests that covers the psychological, social, behavioral, and ethical aspects of cancer. While the *Journal of Experimental & Clinical Cancer Research* takes a more generic approach in cancer research, the other two mentioned journals publish original research with topics more specific to BC. Among the 221 journals analyzed, those with more publications and citations are cancer-specific journals, instead of PA or exercise research publications. This could mean that there are more studies about PA and BC published in cancer journals than in PA, but this phenomenon cannot be confirmed as the Journal Citation Reports (JCR) categories of the journals were not included in the current analysis. Regarding the distribution of scientific production on BC by country, the majority of articles on this topic were produced by a co-author whose institution was from the United States (37.6% of the documents), Canada (10.9%), or China (8.1%) (**Figure 3**). Co-authors from these three countries produced more than half of the articles on this topic. The United States also leads in the category of countries with most co-citations, along with Canada and Germany that follow them in second and third place, respectively. As shown in **Figure 4**, 55 countries/regions collaborated with others, and at least eight significant clusters were formed. The cluster led by the United States stands out among the rest; it has the biggest production numbers and size, and authors from this country published with other institutions from 11 different countries/regions. The cluster led by Australia is also noteworthy, since it has publications with authors from seven different countries and also has great production numbers. In terms of collaboration numbers, Australia and England share second place with 11 collaborations each. Again, the United States is the country leading in this category with more than double the collaborations than the regions in second place (23 total collaborations).

In terms of the authorship of the documents in this topic, 2,690 co-authors shared between 1 and 17 articles. It is observed that there are few authors with several publications on this subject; of the 2,690 co-authors with between 1 and 17 co-publications, 2,265 co-authors had published only one single paper on this subject at the time of analysis. Various analyses were applied to identify the more prominent authors in this field of research, and the number of articles published, citations, and collaborations was examined. According to Lotka’s law, 34 co-authors with five or more articles were identified as the more prolific authors. After applying the h-index, 33 co-authors with at least 33 citations each were found as the more highlighted authors. Several relevant groups of co-authors were identified in this topic; among them, the most outstanding group of researchers was the one led by Courneya, who has collaborations with nine other authors. This author, Courneya, has the most co-citations among the 11,241 co-authors who have been co-cited together in at least one publication about the subject of PA and BC. Within this group, there are 34 noteworthy co-authors who have at least 41 co-citations. In the current analysis, Courneya is established as the most prominent author in PA and BC, since he leads the categories of co-citations and number of collaborations with other authors. Furthermore, he is a co-author of the most relevant article on the topic that was published in 2007 titled “*Effects of aerobic and resistance exercise in breast cancer patients receiving adjuvant chemotherapy: A multicenter randomized controlled trial*” that has, so far, been cited 663 times up to this date.

The article with most citations, “*Effects of aerobic and resistance exercise in breast cancer patients receiving adjuvant chemotherapy: A multicentre randomized controlled trial*” (50), evaluates the relative effect of aerobic and resistance exercise in mitigating unfavorable effects of cancer chemotherapy on physical functioning, body composition, psychosocial, and QoL. This article suggested that aerobic and resistance exercise improved self-esteem, physical fitness, body composition, and chemotherapy completion rate without causing lymphedema or significant adverse events, although no significant improvements in cancer-specific QoL were shown in BC patients receiving chemotherapy. QoL, PA, and exercise have been subjects of interest for many investigators, given the number of other BC-related published articles (53–57, 59).

From the analysis of the most frequently used keywords used in the field of BC and PA research, it can be affirmed that the analyzed body of research focuses on BC, QoL, PA, and exercise. The term breast cancer was the most commonly used term with 266 uses, followed by QoL with 115 uses. Both terms formed a cluster with chemotherapy and cancer survivors; the period of time when these terms were used more was between the years 2015 and 2016. Also, the terms physical activity and survivors formed another cluster with BC survivors and obesity; the higher usage of those terms occurred between 2016 and 2017. In addition, the term exercise formed a cluster of author’s keywords with oncology, cancer, and breast neoplasia; the period when these terms were more actively used was between late 2016 and early 2017. Finally, fatigue, depression, and anxiety formed another cluster, which were predominantly used in 2018.

The present study has some limitations inherent to bibliometric research. Data were taken from WoSCC, so articles indexed in other databases that could have been included were missed. This database is one of the most widely used by researchers for bibliometric analysis (60–62) due to its large catalog, its influence in the scientific field, and the complete information provided for this type of analysis. Information was extracted by bibliometric tools that may lead to bias (63). An analysis of the institutions involved was not carried out, which entails another limitation. Finally, with regard to the inclusion and exclusion criteria, data cleaning was not taken into account, so it cannot be guaranteed that duplicate articles or those that did not meet the criteria were eliminated and should be taken into account in future bibliometric analysis studies.

## 5 Conclusions

This bibliometric analysis provides an overview of the results of BC and PA research worldwide. There has been an exponential growth trend in research on BC and PA since 2001 to 2021. Thus, PA and BC are topics of increasing interest that can be expected to keep gaining momentum. The United States is the country that produces more scientific knowledge on the topic addressed (37.6% of the publications), with the most relevant author being Kerry S. Courneya. In addition, *Psycho-Oncology* is the most attractive journal for BC and PA researchers.

## Author contributions

F-AS was the lead author. D-ZA and J-CR designed and coordinated the study. F-AS and P-CR participated in introduction. D-ZA, P-PD, F-GJ participated in data analysis, article drafting, table/figure creation. F-AS, P-CR, and P-PD participated in discussion. F-AS, D-ZA and J-CR participated in article revision. All authors contributed to the article and approved the submitted version.
