# Supplementary material for: Breast cancer and physical activity: A bibliometric analysis
Source: Front Oncol. 2023 Jan 12;12:1051482. doi: 10.3389/fonc.2022.1051482 (PMC9879290; doi:10.3389/fonc.2022.1051482)
Supplement: Supplementary file 2 [file Table_1.docx]

Supplementary Material

**Supplementary Table 1.**

| Table S1. Bradford core of physical activity and breast cancer journals, by number of journal articles. | | | | | | |  |
| --- | --- | --- | --- | --- | --- | --- | --- |
| Bradford's zone | Journals (Publisher) | JCR | Articles | % Art. | % Acc. | Cites | % O.A. |
| **Core** | Psycho-Oncology (Wiley) | Q1 | 36 | 7.1% | 7.1% | 1792 | 10.5% |
|  | Supportive care in cancer (Springer) | Q2 | 27 | 5.3% | 12.5% | 342 | 14.6% |
|  | Breast cancer research and treatment (Springer) | Q2 | 24 | 4.7% | 17.2% | 841 | 21.4% |
|  | Cancer nursing (Lippincott Williams & Wilkins) | Q1 | 17 | 3.4% | 20.6% | 235 | 3.4% |
|  | BMC Cancer (BMC) | Q2 | 15 | 3.0% | 23.5% | 364 | 99.9% |
|  | Integrative cancer therapies (Sage Publications Inc) | Q2 | 13 | 2.6% | 26.1% | 83 | 99.7% |
|  | International journal of environmental research and public health (MDPI) | Q1 | 11 | 2.2% | 28.3% | 28 | 99.8% |
|  | Journal of cancer survivorship (Springer) | Q1 | 10 | 2.0% | 30.2% | 138 | 19.8% |
|  | Cancer (Wiley) | n.a. | 9 | 1.8% | 32.0% | 445 | n.a. |
|  | Journal of clinical oncology (Lippincott Williams & Wilkins) | Q1 | 8 | 1.6% | 33.6% | 1416 | 15.9% |
|  | Quality of life research (Springer) | Q1 | 8 | 1.6% | 35.2% | 246 | 27.7% |
|  | Trials (BMC) | Q4 | 8 | 1.6% | 36.8% | 67 | 99.8% |
| % Acc. (Percertage of accumulated cites); % O.A. (Percentage of open access). | | | | | | | |
