# Supplementary material for: Breast cancer and physical activity: A bibliometric analysis
Source: Front Oncol. 2023 Jan 12;12:1051482. doi: 10.3389/fonc.2022.1051482 (PMC9879290; doi:10.3389/fonc.2022.1051482)
Supplement: Supplementary file 3 [file Table_2.docx]

Supplementary Material

**Supplementary Table 2.**

| Table S2. Bradford's zones and their number of journals, according to number of articles. | | | | | | | | | | |
| --- | --- | --- | --- | --- | --- | --- | --- | --- | --- | --- |
| Zone | Nº journals (%) | | Number articles (%) | | Acc. nº journals (%) | | Acc. nº articles (%) | | Bradford multipliers | |
| CORE | 12 | (5%) | 186 | (37%) | 16 | (5%) | 113 | (37%) |  | |
| Zone 1 | 56 | (25%) | 167 | (33%) | 59 | (31%) | 214 | (70%) | 0.90 | |
| Zone 2 | 153 | (69%) | 153 | (30%) | 213 | (100%) | 368 | (100%) | 0.92 | |
| Total | 221 | 100% | 506 | 100% |  |  |  |  | Mean | 0.9 |
