# Supplementary material for: Breast cancer and physical activity: A bibliometric analysis
Source: Front Oncol. 2023 Jan 12;12:1051482. doi: 10.3389/fonc.2022.1051482 (PMC9879290; doi:10.3389/fonc.2022.1051482)
Supplement: Supplementary file 4 [file Table_3.docx]

Supplementary Material

**Supplementary Table 3.**

| Table S3. Bradford's zones and their number of journals, according to number of citations. | | | | | | | | | | |
| --- | --- | --- | --- | --- | --- | --- | --- | --- | --- | --- |
| Zone | Nº journals (%) | | Number articles (%) | | Acc. nº journals (%) | | Acc. nº articles (%) | | Bradford multipliers | |
| CORE | 4 | (2%) | 4515 | (33%) | 4 | (2%) | 4515 | (33%) |  | |
| Zone 1 | 17 | (8%) | 4499 | (33%) | 25 | (10%) | 9014 | (66%) | 1.00 | |
| Zone 2 | 200 | (90%) | 4568 | (34%) | 221 | (100%) | 13582 | (100%) | 1.02 | |
| Total | 221 | 100% | 13582 | 100% |  |  |  |  | Mean | 1.0 |
