# Supplementary material for: Breast cancer and physical activity: A bibliometric analysis
Source: Front Oncol. 2023 Jan 12;12:1051482. doi: 10.3389/fonc.2022.1051482 (PMC9879290; doi:10.3389/fonc.2022.1051482)
Supplement: Supplementary file 5 [file Table_4.docx]

Supplementary Material

**Supplementary Table 4.**

| Table S4. Co-authored countries/regions: publications and citations. | | | | | | | |
| --- | --- | --- | --- | --- | --- | --- | --- |
| Position | Country | Documents | Citations | Position | Country | Documents | Citations |
| 1 | Usa | 190 | 5408 | 29 | Norway | 3 | 39 |
| 2 | Canada | 55 | 2465 | 30 | Saudi Arabia | 3 | 66 |
| 3 | Peoples R China | 41 | 421 | 31 | Switzerland | 3 | 176 |
| 4 | Germany | 31 | 1036 | 32 | Cyprus | 2 | 22 |
| 5 | Australia | 28 | 861 | 33 | Malaysia | 2 | 11 |
| 6 | South Korea | 24 | 336 | 34 | Pakistan | 2 | 2 |
| 7 | England | 20 | 796 | 35 | United Arab Emirates | 2 | 6 |
| 8 | Spain | 20 | 446 | 36 | Wales | 2 | 2 |
| 9 | France | 19 | 307 | 37 | Austria | 1 | 36 |
| 10 | Netherlands | 17 | 843 | 38 | Chile | 1 | 5 |
| 11 | Turkey | 15 | 241 | 39 | Colombia | 1 | 1 |
| 12 | Brazil | 14 | 108 | 40 | Ethiopia | 1 | 0 |
| 13 | Italy | 14 | 269 | 41 | Hungary | 1 | 6 |
| 14 | Taiwan | 11 | 151 | 42 | Iceland | 1 | 43 |
| 15 | Denmark | 9 | 234 | 43 | Indonesia | 1 | 0 |
| 16 | Finland | 9 | 214 | 44 | Ireland | 1 | 17 |
| 17 | Iran | 8 | 481 | 45 | Jordan | 1 | 5 |
| 18 | Sweden | 7 | 113 | 46 | Kenya | 1 | 0 |
| 19 | Poland | 6 | 89 | 47 | Lithuania | 1 | 5 |
| 20 | Scotland | 6 | 599 | 48 | Luxembourg | 1 | 1 |
| 21 | Belgium | 5 | 135 | 49 | Nigeria | 1 | 0 |
| 22 | Greece | 5 | 44 | 50 | North Ireland | 1 | 9 |
| 23 | Israel | 4 | 68 | 51 | Oman | 1 | 61 |
| 24 | Japan | 4 | 36 | 52 | Serbia | 1 | 5 |
| 25 | Mexico | 4 | 85 | 53 | Singapore | 1 | 30 |
| 26 | Portugal | 4 | 97 | 54 | Thailand | 1 | 1 |
| 27 | India | 3 | 123 | 55 | Vietnam | 1 | 4 |
| 28 | New Zealand | 3 | 82 |  |  |  |  |
