# Supplementary material for: Breast cancer and physical activity: A bibliometric analysis
Source: Front Oncol. 2023 Jan 12;12:1051482. doi: 10.3389/fonc.2022.1051482 (PMC9879290; doi:10.3389/fonc.2022.1051482)
Supplement: Supplementary file 6 [file Table_5.docx]

Supplementary Material

**Supplementary Table 5.**

| Table S5. Most prolific co-authors. | | | |
| --- | --- | --- | --- |
| Co-Authors name | Main affiliation / Countries/Regions | Articles | Citations |
| Courneya, K. | University of Alberta / Canada | 17 | 1454 |
| Sabiston, C. | University of Toronto / Canada | 11 | 156 |
| McAuley, E. | University of Illinois System / USA | 10 | 308 |
| Rogers, L. | University of Alabama Birmingham / USA | 9 | 332 |
| Arroyo-Morales, M. | University of Granada / Spain | 8 | 207 |
| Cantarero-Villanueva, I. | University of Granada / Spain | 8 | 207 |
| Fernandez-Lao, C | University of Granada / Spain | 8 | 207 |
| Brunet, J. | University of Ottawa / Canada | 7 | 49 |
| Dobos, G. | University of Duisburg Essen / Germany | 7 | 331 |
| Natarajan, L. | University of California San Diego / USA | 7 | 210 |
| Phillips, S. | Northwestern University / USA | 7 | 157 |
| Pinto, B. | University of South Carolina System / USA | 7 | 467 |
| Baumgartner, K. | University of Utah / USA | 6 | 238 |
| Bernstein, L. | University of Connecticut / USA | 6 | 348 |
| Blomqvist, C. | Helsinki University Hospital / Finlad | 6 | 180 |
| Friedenreich, C. | Alberta Health Services / Canada | 6 | 912 |
| Galiano-Castillo, N. | University of Granada / Spain | 6 | 140 |
| Mutrie, N. | University of Edinburgh / Scotland | 6 | 599 |
| Pierce, J. | University of California San Diego / USA | 6 | 212 |
| Saarto, T. | University of Helsinki / Finland | 6 | 180 |
| Stanton, A. | University of California Los Angeles / USA | 6 | 527 |
| Carayol, M. | Serv Protect Maternelle & Infantile / France | 5 | 158 |
| Cramer, H. | University of Technology Sydney / Australia | 5 | 300 |
| Huovinen, R. | University of Turku / Finland | 5 | 175 |
| Kellokumpu-Lehtinen, P. | Tampere University / Finland | 5 | 175 |
| Ligibel, J. | Dana-Faber Cancer Institu / USA | 5 | 13 |
| Mackey, J. | University of Alberta / Canada | 5 | 904 |
| Mckenzie, D. | University of British Columbia / Canada | 5 | 1085 |
| Ninot, G. | University of Montpellier / France | 5 | 158 |
| Paul, A. | University of Duisburg Essen / Germany | 5 | 151 |
| Steindorf, K. | Ruprecht Karls University Heidelberg / Germany | 5 | 245 |
| Utriainen, M. | Helsinki University Central Hospital / Finland | 5 | 180 |
| Vallance, J. | Athabasca University / Canada | 5 | 916 |
| Vehmanen, L. | University of Helsinki / Finland | 5 | 175 |
